# Excess all-cause mortality and COVID-19-related mortality: a temporal analysis in 22 countries, from January until August 2020

**DOI:** 10.1093/ije/dyab123

**Published:** 2021-07-20

**Authors:** Souzana Achilleos, Annalisa Quattrocchi, John Gabel, Alexandros Heraclides, Ourania Kolokotroni, Constantina Constantinou, Maider Pagola Ugarte, Nicoletta Nicolaou, Jose Manuel Rodriguez-Llanes, Catherine Marie Bennett, Ekaterina Bogatyreva, Eva Schernhammer, Claudia Zimmermann, Antonio Jose Leal Costa, Jackeline Christiane Pinto Lobato, Ngibo Mubeta Fernandes, Ana Paula Semedo-Aguiar, Gloria Isabel Jaramillo Ramirez, Oscar Dario Martin Garzon, Laust Hvas Mortensen, Julia A Critchley, Lucy P Goldsmith, Gleb Denissov, Kristi Rüütel, Nolwenn Le Meur, Levan Kandelaki, Shorena Tsiklauri, Joan O’Donnell, Ajay Oza, Zalman Kaufman, Inbar Zucker, Giuseppe Ambrosio, Fabrizio Stracci, Terje P Hagen, Ivan Erzen, Petra Klepac, Pedro Arcos González, Ángel Fernández Camporro, Bo Burström, Nataliia Pidmurniak, Olesia Verstiuk, Qian Huang, Neil Kishor Mehta, Antonis Polemitis, Andreas Charalambous, Christiana A Demetriou

**Affiliations:** 1 Department of Primary Care and Population Health, University of Nicosia Medical School, Nicosia, Cyprus; 2 University of Nicosia Medical School, Nicosia, Cyprus; 3 Department of Basic and Clinical Sciences, University of Nicosia Medical School, Nicosia, Cyprus; 4 European Commission Joint Research Centre, Ispra, Italy; 5 School of Health and Social Development, Deakin University, Melbourne, Australia; 6 Department of Epidemiology, Center for Public Health, Medical University of Vienna, Vienna, Austria; 7 Institute of Studies in Collective Health (IESC), Federal University of Rio de Janeiro, Rio de Janeiro, Brazil; 8 Department of Epidemiology and Biostatistics, Institute of Collective Health (ISC), Fluminense Federal University Niterói, Brazil; 9 National Health Observatory, National Institute of Public Health, Praia, Cape Verde; 10 Nature, Life and Environment Sciences Department, University Jean Piaget of Cape Verde, Praia, Cape Verde; 11 Medicine Faculty, Cooperative University of Colombia, Villavicencio, Colombia; 12 Department of Public Health, University of Copenhagen, Methods and Analysis, Statistics Denmark, Copenhagen, Denmark; 13 Population Health Research Institute, St George's, University of London, London, UK; 14 Department of Registries, National Institute for Health Development, Tallinn, Estonia; 15 Department of Drug and Infectious Diseases Epidemiology, National Institute for Health Development, Tallinn, Estonia; 16 University of Rennes, EHESP, REPERES—EA 7449, Rennes, France; 17 National Center for Disease Control and Public Health, Tbilisi, Georgia; 18 National Statistics Office of Georgia, Tbilisi, Georgia; 19 HSE-Health Protection Surveillance Centre, Dublin, Ireland; 20 Israel Center of Disease Control, Ministry of Health, Ramat Gan, Israel; 21 Faculty of Medicine, Tel Aviv University, Tel Aviv, Israel; 22 Department of Medicine, University of Perugia School of Medicine, Perugia, Italy; 23 CERICLET- Interdepartmental Center for Clinical and Translational Research, University of Perugia School of Medicine, Perugia, Italy; 24 Department of Health Management and Health Economics, University of Oslo, Oslo, Norway; 25 Public Health School, National Institute of Public Health, Ljubljana, Slovenia; 26 Communicable Diseases, National Institute of Public Health, Ljubljana, Slovenia; 27 Department of Medicine, University of Oviedo, Oviedo, Spain; 28 Department of Global Public Health, Karolinska Institutet, Stockholm, Sweden; 29 Faculty of Medicine, Bogomolets National Medical University, Kyiv, Ukraine; 30 SC Center for Rural and Primary Health Care and Department of Geography, University of South Carolina, Columbia, SC, USA; 31 Department of Preventive Medicine and Population Health, University of Texas Medical Branch, Galveston, TX, USA; 32 University of Nicosia, Nicosia, Cyprus

**Keywords:** COVID-19, SARS-CoV-2, pandemic, mortality, infection control

## Abstract

**Background:**

This study aimed to investigate overall and sex-specific excess all-cause mortality since the inception of the COVID-19 pandemic until August 2020 among 22 countries.

**Methods:**

Countries reported weekly or monthly all-cause mortality from January 2015 until the end of June or August 2020. Weekly or monthly COVID-19 deaths were reported for 2020. Excess mortality for 2020 was calculated by comparing weekly or monthly 2020 mortality (observed deaths) against a baseline mortality obtained from 2015–2019 data for the same week or month using two methods: (i) difference in observed mortality rates between 2020 and the 2015–2019 average and (ii) difference between observed and expected 2020 deaths.

**Results:**

Brazil, France, Italy, Spain, Sweden, the UK (England, Wales, Northern Ireland and Scotland) and the USA demonstrated excess all-cause mortality, whereas Australia, Denmark and Georgia experienced a decrease in all-cause mortality. Israel, Ukraine and Ireland demonstrated sex-specific changes in all-cause mortality.

**Conclusions:**

All-cause mortality up to August 2020 was higher than in previous years in some, but not all, participating countries. Geographical location and seasonality of each country, as well as the prompt application of high-stringency control measures, may explain the observed variability in mortality changes.

Key Messages

- Some countries showed excess all-cause mortality between January and August 2020, whereas others displayed either negligible excess mortality or even a decrease in all-cause mortality.
- Excess mortality may be partly attributed to delayed application of strict control measures.
- Lack of excess mortality may be due to seasonality and/or strict control measures in the participating countries.
- Sex-specific mortality was different from total mortality in some countries.
- The synergistic effect of several predictors of mortality warrants investigation.

## Introduction

A new coronavirus, severe acute respiratory syndrome coronavirus 2 (SARS-CoV-2), emerged in late 2019 as a cause of pneumonia in humans. SARS-CoV-2 rapidly spread worldwide from the initial outbreak site in the city of Wuhan, China, leading the World Health Organization (WHO) to declare a global pandemic on 11 March 2020. One year after its identification, the novel coronavirus had infected >80 million individuals and was responsible for >2.04 million deaths, with confirmed cases in 214 countries.^1^

To assess the health burden of the coronavirus disease 2019 (COVID-19) pandemic, the excess mortality (defined as the difference between expected and observed mortality in a given time period) has been investigated and described in several countries.^2–11^ However, the impact of the COVID-19 pandemic on mortality is not completely captured by the analysis of the reported COVID-19 deaths and cases due to limited testing capacity, disruption of health services and a possible reduction in other causes of death as a consequence of restrictive control measures. Indeed, many studies have highlighted that COVID-19 deaths represent only a small proportion of the excess mortality observed since the start of the pandemic in several countries,^5^^,^^6^^,^^12^^,^^13^ indicating that indirect excess deaths may also contribute to the overall mortality burden. This is particularly true in countries heavily affected by the pandemic such as Italy,^5^^,^^6^ France,^6^ Brazil,^14^^,^^15^ the UK^6^ and the USA.^12^^,^^13^ Thus, analysis of overall excess mortality represents an important complementary tool to investigate the influence of the SARS-CoV-2 pandemic on mortality. Importantly, in the case of COVID-19, during the initial weeks of the pandemic, most countries lacked adequate testing and healthcare systems were overwhelmed with patients displaying symptoms of COVID-19; therefore, many cases and deaths that should have been attributed to COVID-19 were not tested and identified.^16–18^ For this reason, all-cause excess deaths (i.e. observed deaths during the pandemic over those expected in the same period of previous years) is recommended by the WHO and the European Centre for Disease Prevention and Control as a more reliable metric for comparing countries/regions.^19^

Excess mortality in countries less affected by COVID-19 and the extent to which any excess can be attributed to COVID-19 are less well researched. In addition, most studies investigating excess mortality to date have focused on single countries or world regions and have mostly relied on publicly available data.^2–11^ Furthermore, existing studies have not interpreted the differences across jurisdictions or over time in the context of COVID-19 control measures and/or death-reporting criteria.^2–11^ This leaves a gap as to the excess-mortality picture in countries without publicly available data and in the interpretation of differences based on factors beyond excess deaths alone.

To this end, an international consortium consisting of >50 institutions across 52 countries and six continents was formed to investigate excess mortality during the COVID-19 pandemic. The consortium attempts to include countries worldwide without restriction and constitutes an ongoing effort to monitor overall and cause-specific mortality resulting from the COVID-19 pandemic. The present study investigates overall and sex-specific excess all-cause mortality since the inception of the COVID-19 pandemic until August 2020 in 22 countries.

## Methods

### Data acquisition

In this study, we examined the mortality data from 22 countries participating in the international consortium (Supplementary Figure S1 and Table S1, available as Supplementary data at *IJE* online) that have collected and provided data until the end of either June (*n* = 5) or August 2020 (*n* = 17), depending on data availability. Information was collected for total and sex-specific all-cause mortality (for 2015–2020), as well as total and sex-specific COVID-19 deaths (for 2020). Anonymous data were collected from national vital-statistics databases, either publicly available or with restricted access, from each participating country to the latest available data point of 2020 (Supplementary Table S1, available as Supplementary data at *IJE* online).

Countries reported all-cause mortality and COVID-19 deaths by week [either International Organization for Standardization (ISO) week, starting on Monday; or epidemiological (Epi) week, starting on Sunday; or another national counting week system, depending on the country]. COVID-19-death reporting also differed between countries, as shown in Supplementary Table S1, available as Supplementary data at *IJE* online. Some countries (*n* = 11) reported as COVID-19 deaths any deaths among positive cases irrespective of where COVID-19 was listed on the death certificate. Thus, COVID-19 was either listed among the chain of causes leading to death or as a contributing condition on the death certificate [cause of death (COD) or contributing condition].^20^ Other countries (*n* = 11) reported as COVID-19 deaths only the deaths for which COVID-19 was listed among the chain of causes leading to death (COD);^20^ of these latter countries, France reported only hospital and nursing-home COVID-19 deaths. The national primary data sources used in this study and endorsed by the national partners might differ from publicly available repositories/databases, primarily due to the retrospective addition of cases and deaths declared with some delay. Data were collected during October and November 2020, several weeks after the end of the study period, to account for reporting delays (ranging from a few days to a few weeks).^6^^,^^21^^,^^22^ The national data source, the period of available mortality data, time units and COVID-19-death definitions used per country are summarized in Supplementary Table S1, available as Supplementary data at *IJE* online.

### Statistical analysis

Excess mortality for 2020 was calculated by comparing weekly or monthly 2020 mortality (observed deaths) against a baseline mortality obtained from 2015–2019 data for the same week or month using two different methods. The choice of 5 years for the baseline mortality estimation was based on widely adopted practices from well-established surveillance consortia^3^^,^^23^^,^^24^ as well as on other published studies.^2^ In the first method, the baseline was computed as the average mortality rate of the previous 5 years.^8^ In the second method, the baseline was estimated based on historical data accounting for seasonality and long- and short-term trends, representing the expected number of deaths in 2020.^24–26^

### Method 1: Observed 2020 vs 2015–2019 average mortality rates

For each country (*n* = 22) and year, the weekly or monthly observed number of deaths was divided by the country’s population at the beginning of the particular year to obtain weekly and monthly mortality rates. Therefore, country-specific populations were assumed to be constant throughout the year and mortality rates were expressed as deaths per 100 000 population. Mortality rates instead of number of deaths were used to account for population differences. Total and sex-specific population estimates for the participating countries were obtained from the World Bank,^27^ except for the UK nations, for which data from the Office for National Statistics^28^ were used, and for Cyprus, for which Eurostat data^29^ were used to include only the population in the Republic of Cyprus government-controlled area. Population estimates for 2019 were also applied to 2020 data. For each country, the average weekly or monthly total and sex-specific mortality rate for 2015–2019 was calculated and plotted against weekly or monthly 2020 mortality rates up to the latest available data point of 2020, to provide a visual representation of excess mortality by time point. For data visualization, we downloaded and used a stringency index (SI)—a composite measure based on nine response indicators including school closures, workplace closures and travel bans, rescaled to a value from 0 to 100 (100 = strictest), from the Oxford COVID-19 Government Response Tracker.^30^ Although the index should not be interpreted as a score for the appropriateness or effectiveness of a country’s response, it provides an indication of the number and strictness of government policies. For each country and time unit of 2020, the SI was categorized as low (<25%), moderate (between 25% and 74%) and high (≥75%). The SI categories were then plotted together with the country profiles of excess mortality.

Then, the average year-to-date (YTD) mortality rate (mortality rate up to the final week, month or trimester of data report for each country) between 2015 and 2019 was considered as the ‘baseline’, whereas the excess mortality for 2020 was estimated for each country by subtracting the baseline from the 2020 YTD mortality rate. For Sweden and Cape Verde, only monthly data were available for this analysis. For Colombia, trimester data were used for the YTD comparison (trimester data could not be graphically compared). For all other countries, weekly data were used for the graphical and YTD comparisons.

### Method 2: Observed vs expected 2020 deaths

The expected number of deaths for 2020 was modelled using Poisson regression, assuming a quasi-Poisson distribution to account for overdispersion in the weekly mortality counts. We used a Generalized Linear Model with a linear time trend (weekly mortality as the time unit) to adjust for secular trends and two sine and cosine terms for yearly and half-yearly seasonal cycles. The terms for sine-type cyclical seasonality were chosen based on the weekly distribution of the data and periodograms. Other periodicities (i.e. 4 and 9 months) were also tested but were omitted because the effect was negligible and did not improve the model fit. The same model was applied to all countries with weekly data, separately. The regression models were built on complete weeks and any truncated weeks were excluded. Truncated weeks were observed during the last week of the year in Australia, England and Wales, Scotland and during the last 2 weeks in Northern Ireland. Different death counts around Christmas and New Year were observed and accounted for.^2^ The residual variation was corrected for skewness by applying a 2/3 power transformation.^25^ The 95% confidence intervals (CIs) and standard deviation for expected deaths were also estimated. The weekly results of the observed vs expected deaths are displayed graphically for each country using z-scores [(number of observed deaths – expected mortality)/standard deviation of the residuals]. Z-scores that range between –2 and +2 are considered ‘normal’ and a value of >4 Z-scores is considered a substantial increase.^3^^,^^31^

Then, the sum of the expected 2020 deaths was subtracted from the sum of the observed 2020 deaths to obtain an estimate of excess deaths. The statistical significance of excess deaths was determined using the 95% CIs estimated by the model.

It is important to note that only countries providing weekly data (*n* = 19) were included in the second methodological approach. In addition, 2019 weekly mortality data were not available for Scotland and therefore the estimation of ‘baseline’ was based on 2015–2018 data in both methods. For Northern Ireland and Spain, sex-specific all-cause mortality data were not available at the time of data analysis, so sex-specific all-cause mortality was calculated for 17 countries. England and Wales were considered as one country for the purposes of analysis and reporting of results, as combined data are routinely provided this way. For each country, the reported type of COVID-19 death was used in both methods and no between-country comparisons were attempted. A sensitivity analysis involving the truncation of the observation period to week 26 (June 2020) for all countries was conducted to investigate any further reporting delay.

All analyses were performed in R Statistical Software, version 3.6.1 (the R Foundation for Statistical Computing, Vienna, Austria).

## Results

### Observed 2020 vs 2015–2019 average mortality rates

Tables 1 and 2 compare the country-specific YTD mortality rates of the previous 5 years (2015–2019) to 2020 for the total population and by sex, respectively. The total all-cause mortality rate (total, males and females) was higher during 2020 compared with the average of the previous 5  years in 11 out of the 22 participating countries: Brazil, Cyprus, England and Wales, France, Italy, North Ireland, Scotland, Spain, the USA, Slovenia and Sweden. Among the total population, the highest increase, in descending order, was observed for England and Wales, Spain, the USA, Scotland, Brazil and Northern Ireland (>50 deaths per 100 000 population). In France, Sweden, Slovenia, Italy and Cyprus, the increase was less pronounced (<35 deaths per 100 000 population; countries listed in descending order). Within the countries with higher 2020 mortality rates, COVID-19 was reported as a COD in five countries (Cyprus, England and Wales, France, Italy and the USA) and as a COD or contributing condition in the other six countries (Brazil, Northern Ireland, Scotland, Slovenia, Spain and Sweden).

**Table 1:** Excess mortality: observed 2020 mortality rate vs 2015–2019 average mortality rate (deaths per 100 000 population) and COVID-19 mortality rate

Country	Time unit^a^	Time frame: time unit 1 to time unit #	YTD mortality rate (average 2015–2019)	YTD mortality rate (2020)	Difference in mortality rates (2020 vs 2015–2019)	COVID-19 mortality rate
Australia	National week	26	274.9	272.0	–3.0	0.4^††^
Austria	ISO	35	627.0	633.6	6.6	8.2^†^
Brazil	Epi	35	428.6	496.8	68.2	59.1^†^
Cyprus	ISO	35	472.0	487.2	15.1	2.3^††^
Denmark	Epi	35	629.7	621.0	8– .8	10.7^†^
England and Wales	National week	35	617.8	720.5	102.8	89.4^††^
Estonia	ISO	35	601.9	589.3	−12.5	4.7^††^
France	ISO	35	616.1	649.8	33.7	30.0^††^
Georgia	ISO	35	874.7	810.6	–64.1	0.5^††^
Ireland	ISO	26	330.9	331.5	0.6	29.8^††^
Israel	Epi	35	353.1	354.4	1.3	10.1^†^
Italy	Epi	26	555.9	574.2	18.3	57.6^†^
Northern Ireland	National week	35	567.7	620.4	52.8	47.3^††^
Norway	ISO	35	521.1	506.3	–14.8	4.9^†^
Scotland^b^	ISO	35	721.8	808.2	86.4	78.7^††^
Slovenia	ISO	35	659.1	678.8	19.7	6.1^†^
Spain	Epi	35	614.0	713.0	99.0	70.8^†^
Ukraine	ISO	35	853.8	852.3	–1.5	7.5^††^
USA	Epi	35	578.2	666.6	88.3	56.9^†^
Cape Verde	Month	6	254.8	237.3	–17.5	2.7^†^
Colombia	Trimester	2	229.8	230.1	0.3	8.5^†^
Sweden	Month	8	594.2	619.4	25.1	56.3^††^

YTD, mortality rate up to the final week/month/trimester of data report for each country; ISO, International Organization for Standardization week; Epi, epidemiological week.

a ISO week: Monday–Sunday; Epi week: Sunday–Saturday; National week: Australia uses 7 days starting from 1 January; England, Wales and Northern Ireland use Saturday–Friday.

b For Scotland, baseline comparison was the average of years 2015–2018, as 2019 data were not available.

† COVID-19 was a cause of death (COD) or a contributing factor to death.

†† COVID-19 was a COD.

**Table 2:** Excess mortality by sex: observed 2020 mortality rate vs 2015–2019 average mortality rate (deaths per 100 000 population) and COVID-19 mortality*^§^

Country	Time unit^a^	Time frame: time unit 1 to time unit #	Males				Females			
			YTD mortality rate (average 2015–2019)	YTD mortality rate (2020)	Difference in mortality rates (2020 vs 2015–2019)	COVID-19 mortality rate	YTD mortality rate (average 2015–2019)	YTD mortality rate (2020)	Difference in mortality rates (2020 vs 2015–2019)	COVID-19 mortality rate
Australia	National week	26	274.8	274.8	0.0		275.1	269.2	–5.9	
Austria	ISO	35	612.8	630.5	17.7		640.6	636.6	–4.1	
Brazil	Epi	35	486.3	570.6	84.3	69.8^†^	372.3	425.5	53.1	48.8^†^
Cyprus	ISO	35	508.0	522.4	14.4	3.3^††^	437.8	453.5	15.7	1.3^††^
Denmark	Epi	35	636.7	638.0	1.4		622.9	604.1	–18.8	
England and Wales	National week	35	614.9	736.1	121.2	99.7^††^	620.6	705.4	84.8	79.4^††^
Estonia	ISO	26	601.7	588.0	–13.7	4.8^††^	602.0	590.5	–11.5	4.7^††^
France	ISO	35	636.2	673.1	36.9	36.3^††^	597.3	628.1	30.8	23.8^††^
Georgia	ISO	35	938.0	871.9	–66.1	0.5^††^	816.9	754.7	–62.2	0.5^††^
Ireland	ISO	26	342.2	339.7	–2.5	29.5^††^	319.7	323.4	3.7	30.0^††^
Israel	Epi	35	358.5	362.7	4.2	10.8^†^	347.7	346.2	–1.5	9.4^†^
Italy	Epi	26	545.7	571.2	25.5		565.6	577.1	11.4	
Norway	ISO	35	500.6	493.8	–6.8		541.9	519.1	–22.8	
Scotland^b^	ISO	35	718.5	830.7	112.2	80.4^††^	725.0	786.9	61.9	77.1^††^
Slovenia	ISO	35	652.5	671.9	19.4	4.8^†^	665.5	685.6	20.0	7.4^†^
Ukraine	ISO	35	920.6	1048.5	127.9	10.7^††^	796.2	683.0	–113.2	4.7^††^
USA	Epi	35	658.5	710.6	52.0	62.2^†^	499.6	623.4	123.8	51.7^†^
Cape Verde	Month	6	282.3	264.8	–17.4	2.5^†^	227.0	209.6	–17.5	2.9^†^
Colombia	Trimester	2	258.2	260.4	2.2	10.7^†^	202.3	200.6	–1.7	6.3^†^
Sweden	Month	8	575.2	614.2	38.9	60.7^††^	613.2	624.6	11.3	52.0^††^

YTD, mortality rate up to the final week/month/trimester of data report for each country; ISO, International Organization for Standardization week; Epi, epidemiological week.

* Empty cells indicate that data were not available for the specific country.

§ Northern Ireland and Spain not included because sex-specific data were not available.

a ISO week: Monday–Sunday; Epi week: Sunday–Saturday; National week: Australia uses 7 days starting from 1 January; England, Wales and Northern Ireland use Saturday–Friday.

b For Scotland, baseline comparison was the average of years 2015–2018, as 2019 data were not available.

† COVID-19 was a cause of death (COD) or a contributing factor to death .

†† COVID-19 was a COD .

By contrast, Cape Verde, Estonia, Georgia and Norway had a reduced YTD mortality rate in 2020 compared with the previous 5 years (range between –12.5 and –64.1 deaths per 100 000 population).

In the rest of the participating countries (Australia, Austria, Colombia, Denmark, Ireland, Israel and Ukraine), we observed discordant results in the mortality-rate changes within the two sexes.

### Weekly/monthly 2020 mortality rates and COVID-19 control measures

**Figure 1:**
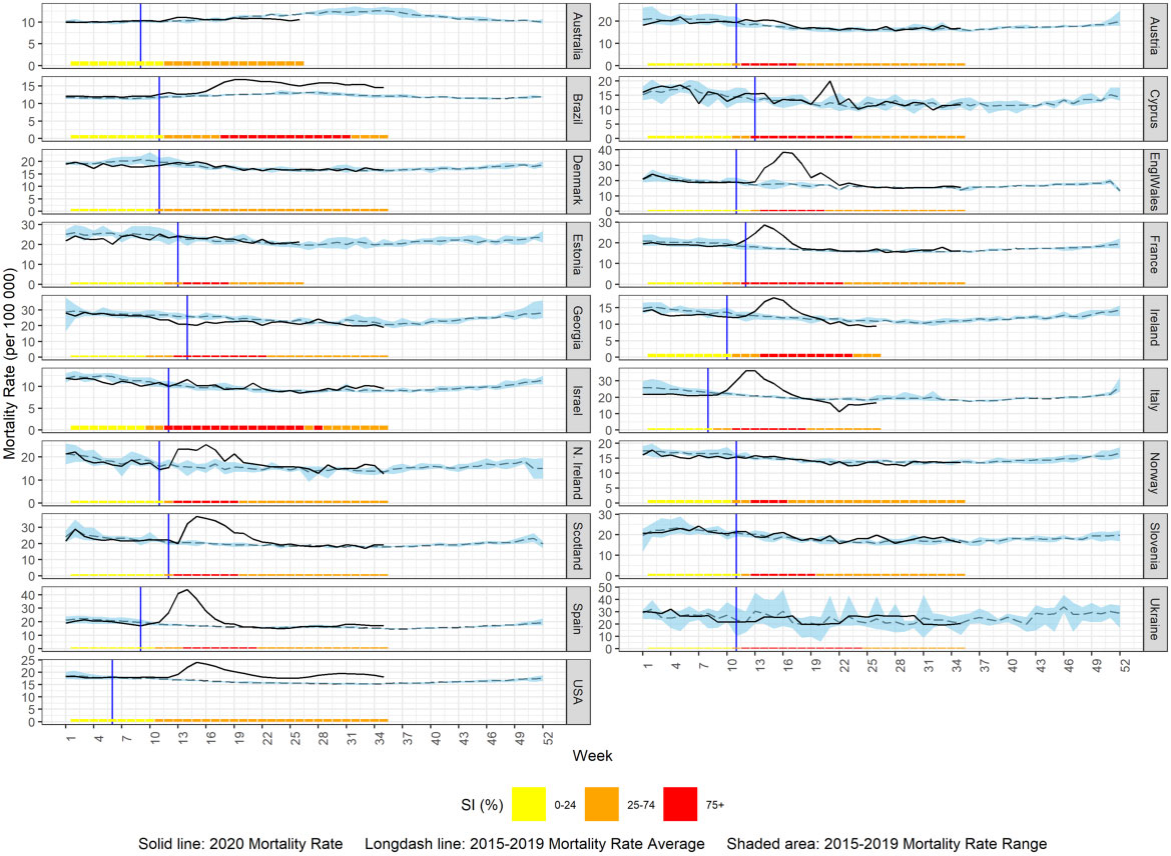
Observed 2020 mortality rate vs 2015–2019 average mortality rate (per 100 000 population) and stringency index (SI, %) for countries providing weekly data (solid vertical line indicates the start of the reported COVID-19 deaths)

**Figure 2:**
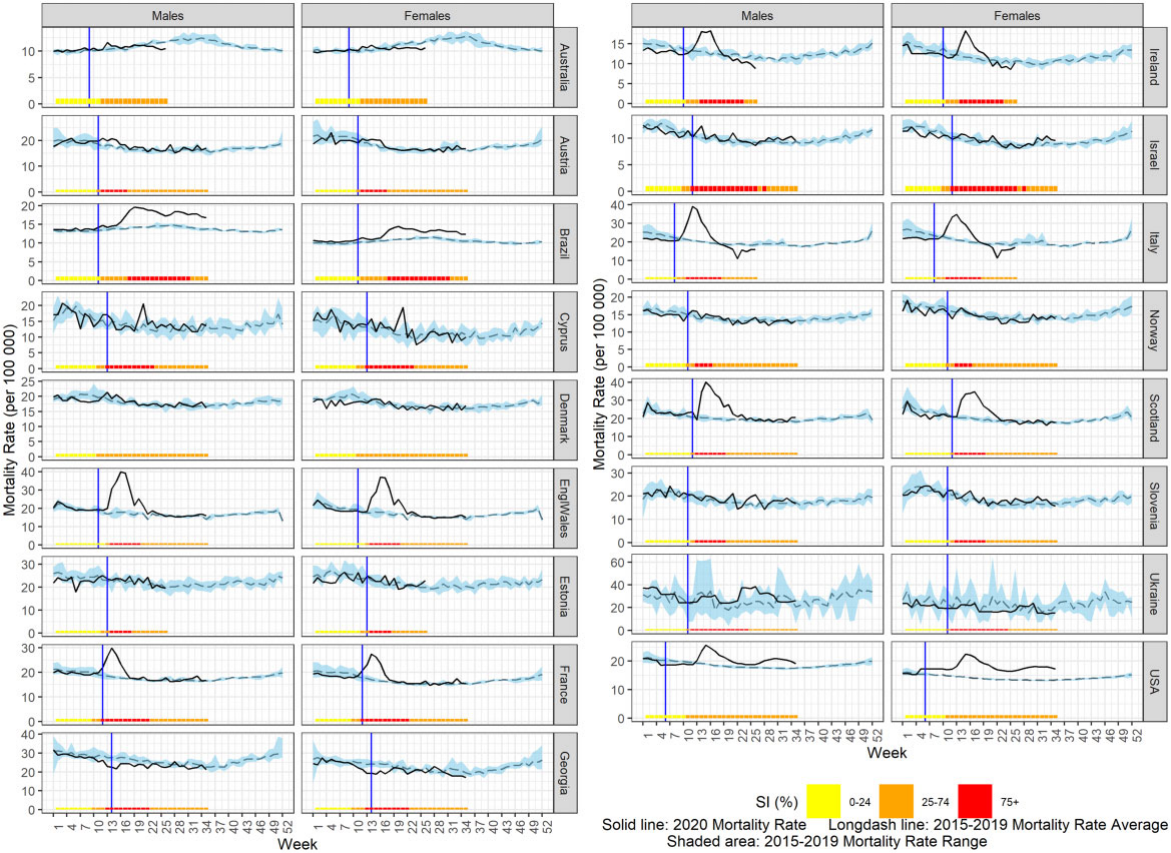
Observed 2020 mortality rate vs 2015–2019 average mortality rate (per 100 000 population) and stringency index (SI, %) by sex for countries providing weekly data (solid vertical line indicates the start of the reported COVID-19 deaths)

**Figure 3:**
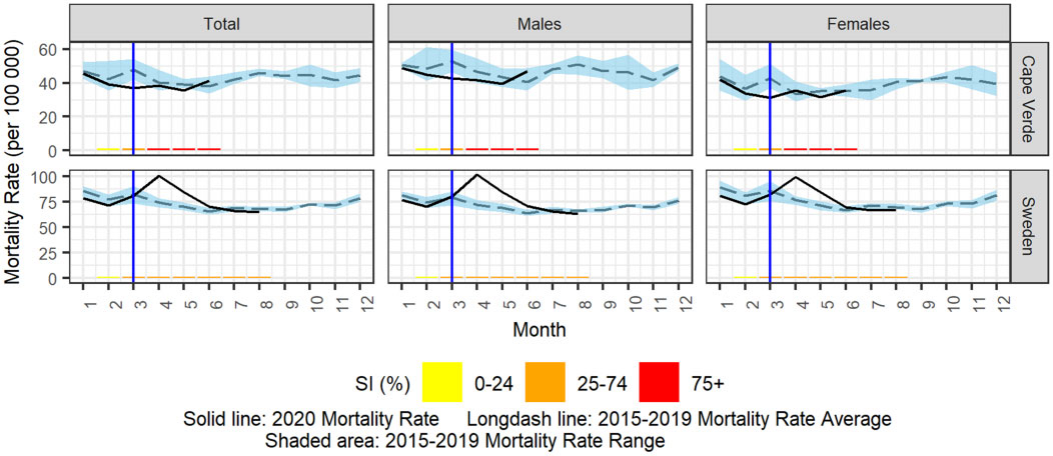
Observed 2020 mortality rate vs 2015–2019 average mortality rate (per 100 000 population) and stringency index (SI, %) for total population and by sex for countries providing monthly data (solid vertical line indicates the start of the reported COVID-19 deaths)

The 2020 all-cause mortality rate against the 2015–2019 average mortality rate per week is displayed graphically for each country using country-specific scales in Figure 1 for total population and Figure 2 by sex. Figure 3 displays countries reporting monthly data; trimester rates were not plotted. The same figures also display the progress of the control measures in each country using the weekly or monthly SI and the onset of COVID-19 death reports.

As shown in Figures 1–3, an excess-mortality rate during 2020 was observed for Brazil, England and Wales, France, Ireland, Italy, Northern Ireland, Scotland, Spain, Sweden and the USA. The maximum mortality rate was observed sometime between week 12/2020 (Italy; 36.2 deaths per 100 000 population) and weeks 19–20/2020 (Brazil; 17.0 deaths per 100 000 population), and in April 2020 in Sweden (100.8 deaths per 100 000 population). The mortality-rate peaks for 2020 were observed at the same time for males and females in France, England and Wales, the USA and Sweden. However, small peaks of excess mortality were observed in Australia (week 13/2020), Austria (weeks 12 and 15/2020), Cyprus (weeks 20–21/2020), Estonia (week 17/2020), Georgia (week 28/2020), Israel (weeks 14, 21 and 34/2020), Slovenia (week 26/2020) and Ukraine (weeks 4, 17 and 29/2020).

COVID-19 deaths were first reported in March 2020 for all the participating countries except in the case of Spain, Italy and the USA, for which COVID-19 deaths were first reported in February 2020 (Figures 1–3). In Australia, deaths due to COVID-19 started at the end of February to the beginning of March and, in Georgia, in late March to early April 2020. In Colombia, COVID-19 deaths were also first reported during the first trimester of 2020. At the same time, in all countries, the beginning of the implementation of moderate- or high-stringency control measures ranged from week 9 to week 13 (SI: 25–74%) and from week 11 to week 18 (SI: ≥75%).

### Observed vs expected 2020 deaths

For most countries in which all-cause deaths in 2020 (January–June/August) were higher than expected, mortality was raised for both males and females (Brazil, England and Wales, France, Scotland and the USA) (Tables 3 and 4). For Ukraine and Israel, only male deaths were elevated; for Ireland, total and female deaths were elevated; and for Italy, total and male deaths were elevated. For some countries (Northern Ireland and Spain), only total deaths were elevated.

**Table 3:** Excess deaths: observed vs expected 2020 deaths

Country	Time unit^a^	Time frame: week 1 to week #	E deaths (95% CI)	O deaths (2020)	Ratio of O/E deaths	Excess deaths (95% CI)^b^^/c^	COVID-19 deaths
Australia	National week	26	71 068 (70 138; 72 003)	68 985	0.97	–2083 (–3018; –1153)^c^	104^††^
Austria	ISO	35	55 725 (54 553; 56 905)	56 245	1.01	520 (–660; 1692)	728^†^
Brazil	Epi	35	928 308 (920 863; 935 772)	1 048 528	1.13	120 220 (112 756; 127 665)^b^	120 814^†^
Cyprus	ISO	35	4260 (4098; 4423)	4267	1.00	7 (–156; 169)	20^††^
Denmark	Epi	35	37 470 (36 915; 38 028)	36 131	0.96	–1339 (–1897; –784)^c^	624^†^
England and Wales	National week	35	363 219 (354 828; 371 675)	417 088	1.15	53 869 (45 413; 62 260)^b^	51 740^††^
Estonia	ISO	26	7954 (7732; 8178)	7818	0.98	–136 (–360; 86)	63^††^
France	ISO	35	422 087 (415 175; 429 036)	435 787	1.03	13 700 (6751; 20 612)^b^	20 099^††^
Georgia	ISO	35	30 997 (30 193; 31 808)	30 158	0.97	–839 (–1650; –35)^ c^	18^††^
Ireland	ISO	26	15 975 (15 610; 16 342)	16 380	1.03	405 (38, 770)^b^	1471^††^
Israel	Epi	35	31 577 (31 061; 32 096)	32 086	1.02	509 (–10; 1025)	912^†^
Italy	Epi	26	335 133 (326 803; 343 533)	346 236	1.03	11 103 (2703; 19 433)^b^	34 751^†^
Northern Ireland	National Week	35	10 663 (10 303; 11 027)	11 488	1.08	825 (461; 1185)^b^	876^††^
Norway	ISO	35	27 423 (26 973; 27 875)	27 076	0.99	–347 (–799; 103)	264^†^
Scotland^d^	ISO	35	39 512 (38 563; 40 468)	43 423	1.10	3911 (2955; 4860)^b^	4229^††^
Slovenia	ISO	35	14 412 (14 037; 14 789)	14 172	0.98	–240 (–617; 135)	128^†^
Spain	Epi	35	287 014 (281 525; 292 537)	335 657	1.17	48 643 (43 120; 54 132)^b^	33 340^†^
Ukraine	ISO	35	370 875 (337 716; 405 053)	378 306	1.02	7431 (–26 747; 40 590)	3322^††^
USA	Epi	35	1 956 566 (1 940 797; 1 972 377)	2 187 893	1.12	231 327 (215 516; 247 096)^b^	186 754^†^

E, expected; O, observed; ISO, International Organization for Standardization week; Epi, epidemiological week.

a ISO week: Monday–Sunday; Epi week: Sunday–Saturday; National week: Australia uses 7 days starting from 1 January; England, Wales and Northern Ireland use Saturday–Friday.

b Statistically significant increase in 2020 observed deaths compared with expected.

c Statistically significant decrease in 2020 observed deaths compared with expected.

d For Scotland, baseline comparison was the average of years 2015–2018, as 2019 data were not available.

† COVID-19 was a cause of death (COD) or a contributing factor to death.

†† COVID-19 was a COD.

**Table 4:** Excess deaths by sex: observed vs expected 2020 deaths*^§^

Country	Time unit^a^	Time frame: week 1 to week #	E deaths (95% CI)	O deaths (2020)	Ratio O/E deaths	Excess deaths (95% CI)^b^^/c^	COVID-19 deaths
					Males		
	
Australia	National week	26	35 833 (35 325; 36 344)	34 709	0.97	–1124 (–1635; –616)^c^	
Austria	ISO	35	27 082 (26 507; 27 662)	27 560	1.02	478 (–102; 1053)	
Brazil	Epi	35	511 928 (507 710; 516 158)	591 948	1.16	80 020 (75 790; 84 238)^b^	70 135^†^
Cyprus	ISO	35	2238 (2134; 2344)	2235	1.00	–3 (–109; 101)	14^††^
Denmark	Epi	35	19 092 (18 748; 19 438)	18 458	0.97	–634 (–980; –290)^c^	
England and Wales	National week	35	182 453 (178 534; 186 401)	210 180	1.15	27 727 (23 779; 31 646)^b^	28 466^††^
Estonia	ISO	26	3707 (3573; 3839)	3687	1.00	–18 (–152; 114)	30^††^
France	ISO	35	210 169 (207 011; 213 343)	218 467	1.04	8298 (5124; 11 456)^b^	11 795^††^
Georgia	ISO	35	15 890 (15 476; 16 308)	15 468	0.97	–422 (–840; –8)^c^	8^††^
Ireland	ISO	26	8347 (8140; 8557)	8328	1.00	–19 (–229; 188)	724^††^
Israel	Epi	35	15 930 (15 616; 16 246)	16 332	1.03	402 (86; 716)^b^	486^†^
Italy	Epi	26	160 293 (156 716; 163 897)	167 581	1.05	7288 (3684; 10 865)^b^	
Norway	ISO	35	13 405 (13 137; 13 674)	13 338	1.00	–67 (–336; 201)	
Scotland^d^	ISO	35	19 411 (18 956; 19 870)	21 686	1.12	2275 (1816; 2730)^b^	2098^††^
Slovenia	ISO	35	7138 (6913; 7366)	6983	0.98	–155 (–383; 70)	50^†^
Ukraine	ISO	35	188 961 (166 282; 212 586)	215 579	1.14	26 618 (2993; 49 297)^b^	2209^††^
USA	Epi	35	1 111 443 (1 102 159; 1 120 753)	1 154 095	1.04	42 653 (33 343; 51 936)^b^	101 081^†^
					Females		
	
Australia	National week	26	35 241 (34 683; 35 802)	34 276	0.97	–965 (–1526; –407)^c^	
Austria	ISO	35	28 646 (27 957; 29 339)	28 685	1.00	39 (–654; 728)	
Brazil	Epi	35	416 071 (412 427; 419 726)	456 580	1.10	40 509 (36 854; 44 153)^b^	50 661^†^
Cyprus	ISO	35	2021 (1915; 2130)	2032	1.01	11 (–98; 117)	6^††^
Denmark	Epi	35	18 379 (18 050; 18 711)	17 673	0.96	–706 (–1038; –377)^c^	
England and Wales	National week	35	180 815 (176 217; 185 452)	206 908	1.14	26 093 (21 456; 30 691)^b^	23 274^††^
Estonia	ISO	26	4250 (4106; 4395)	4131	0.97	–119 (–264; 25)	33^††^
France	ISO	35	211 921 (207 998; 215 868)	217 320	1.03	5399 (1452; 9322)^b^	8229^††^
Georgia	ISO	35	15 107 (14 630; 15 588)	14 690	0.97	–417 (–898; 60)	10^††^
Ireland	ISO	26	7629 (7395; 7866)	8052	1.06	423 (186; 657)^b^	747^††^
Israel	Epi	35	15 647 (15 316; 15 980)	15 754	1.01	107 (–226; 438)	426^†^
Italy	Epi	26	174 840 (169 978; 179 747)	178 655	1.02	3815 (–1092; 8677)	
Norway	ISO	35	14 018 (13 712; 14 327)	13 738	0.98	–280 (–589; 26)	
Scotland^d^	ISO	35	20 102 (19 526; 20 684)	21 737	1.08	1635 (1053; 2211)^b^	2131^††^
Slovenia	ISO	35	7274 (7047; 7503)	7189	0.99	–85 (–314; 142)	78^†^
Ukraine	ISO	35	182 010 (159 894; 205 061)	162 727	0.89	–19 283 (–42 334; 2833)	1113^††^
USA	Epi	35	845 188 (838 507; 851 887)	1 033 798	1.22	188 609 (181 910; 195 291)^b^	85 673^†^

E: expected; O: observed; ISO, International Organization for Standardization week; Epi, epidemiological week.

* Empty cells indicate that data were not available for the specific country.

§ Northern Ireland and Spain not included because sex-specific data were not available .

a ISO week: Monday–Sunday; Epi week: Sunday–Saturday; National week: Australia uses 7 days starting from 1 January; England, Wales and Northern Ireland use Saturday–Friday.

b Statistically significant increase in 2020 observed deaths compared with expected.

c Statistically significant decrease in 2020 observed deaths compared with expected.

d For Scotland, baseline comparison was the average of years 2015–2018, as 2019 data were not available.

† COVID-19 was a cause of death (COD) or a contributing factor to death.

†† COVID-19 was a COD.

On the contrary, all-cause 2020 deaths in Australia and Denmark (both sexes) and only total and male deaths in Georgia were lower than expected.

The weekly COVID-19 deaths in relation to excess deaths are displayed graphically in Supplementary Figure S2, available as Supplementary data at *IJE* online.

### Weekly deaths Z-score

**Figure 4:**
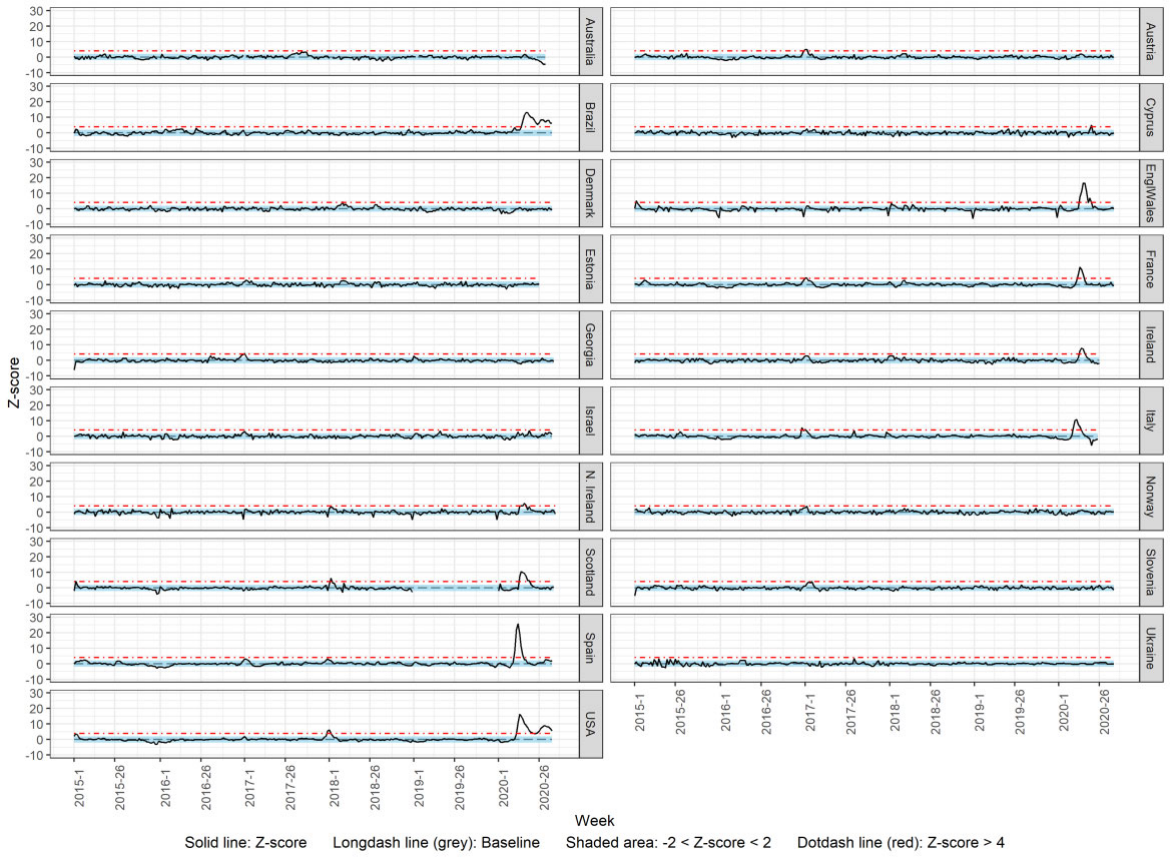
Observed (z-score) vs expected (baseline) deaths

Figure 4 shows the weekly deaths Z-score over time from week 1/2018 to week 26/2020 or week 35/2020, for the total population. The countries that showed a substantial increase (>4 Z-scores) in the observed mortality during 2020 include Brazil, Cyprus, England and Wales, France, Ireland, Italy, Northern Ireland, Scotland, Spain and the USA. The first substantial weekly excess in all-cause deaths was observed in different weeks of 2020 depending on the country; from week 11 (Italy) to week 21 (Cyprus). This excess in mortality lasted from 1 (Cyprus) to 7 (England and Wales, and Scotland) weeks. In Brazil and the USA, a substantial excess mortality has been observed since weeks 17 and 14 of 2020, respectively.

**Figure 5:**
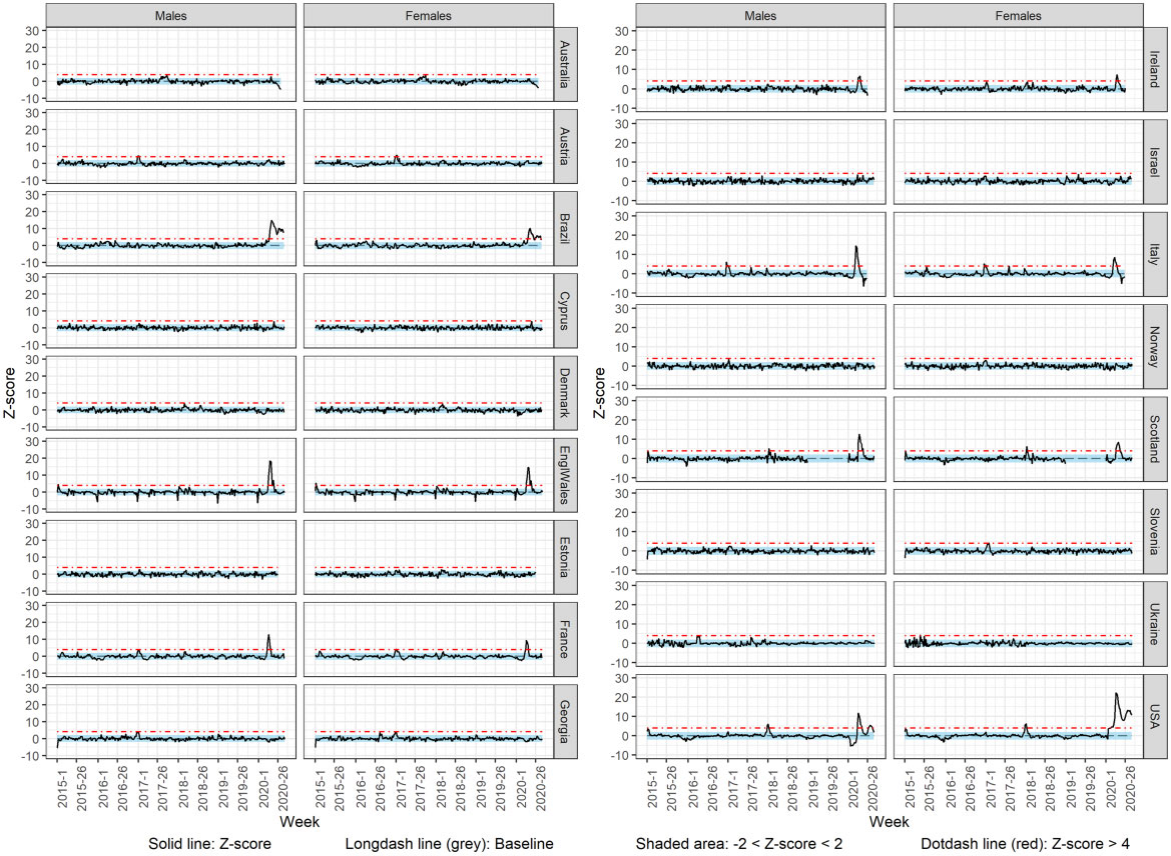
Observed (z-score) vs expected (baseline) deaths by sex

Among the countries with a substantial excess of mortality for the total population, a similar excess was observed for both males and females in Brazil, France, Ireland and the USA, with differences in the duration and timing of observed weekly excess mortality (Figure 5). However, a substantial excess in mortality was observed only for the total population in Cyprus, only for the total population and males in England and Wales, and only for the total population and females in Italy and Scotland.

The sensitivity analysis by truncating the observation period to week 26 for all countries did not show any difference for the participating countries experiencing the first wave of the pandemic in the first half of 2020 (data not shown).

## Discussion

### Summary of findings

In this investigation of 22 countries across five continents, we show that Brazil, France, Ireland, Italy, Spain, Sweden, the UK (England, Wales, North Ireland and Scotland) and the USA demonstrated excess all-cause deaths between January and June or August 2020, among males and females combined. In Italy, the excess deaths were driven by excess deaths among males and, in Ireland, by excess deaths in females. On the other hand, we show that Australia, Denmark and Georgia actually experienced a decrease in deaths in 2020 among males and females combined. Austria, Cyprus, Ireland, Israel, Norway, Slovenia, Ukraine, Cape Verde and Colombia experienced none or only very limited excess deaths among males and females combined.

Our findings on excess mortality in Brazil, France, Italy, Spain, Sweden, the UK (England, Wales, North Ireland and Scotland) and the USA are in agreement with previous publications and reports.^2^^,^^3^^,^^5–8^^,^^10^^,^^11^^,^^13^^,^^32^^,^^33^ Similarly, the lack of an increase in overall all-cause mortality in Australia, Austria, Denmark, Estonia, Israel and Norway was in agreement with previous reports.^2^^,^^3^^,^^32–34^ Colombia was elsewhere demonstrated to have excess mortality during 2020,^35^ but the increase in mortality started towards the end of the observation period of Colombia for this study, explaining the lack of an increase in our results. To our knowledge, this is the first published analysis on excess mortality in Cyprus, Georgia, Ireland, Slovenia, Ukraine and Cape Verde.

### Mortality burden across countries

In several of the participating countries, moderate- and high-stringency control measures were first put in place on the same week or 1 week after the first reported COVID-19 deaths. Other countries implemented such measures ahead of the peak of the pandemic. Still, in some countries, strict control measures were either applied with delay or not applied at all. As discussed below, the mortality burden observed in the participating countries of this study seems to be, at least partly, related to the promptness in the application of control measures of high SI.

The magnitude of excess mortality observed in Brazil, France, Italy, Spain, Sweden, the UK and the USA appears to correspond to lack of, or delay in, the application of strict control measures by the respective governments after the first COVID-19 death in these countries. Italy, Spain, the UK and Brazil enforced high-stringency measures after 3 (Italy, the UK), 5 (Spain) and 7 (Brazil) weeks from the first COVID-19 death. Although, for France, it appears that strict measures were implemented soon after the first rise in COVID-19 deaths in hospitals and nursing homes (week 12), the first COVID-19 deaths occurred in mid-February in the country,^36^ suggesting a delay in enforcing strict control measures. Similarly, in the countries of the UK, the delay between the first death and the application of measures with an SI of ≥75% is likely to be longer due to limited testing taking place in the UK during the first weeks of the pandemic.^37^ In Brazil, pre-existing inequalities in healthcare access, particularly to critically ill patients, also accounted for excess mortality.^38^ On the other hand, Swedish and US governments did not apply measures whose SI was >75% for the duration of this study and this may have contributed to poorer control of the pandemic and higher excess mortality.

Ireland enforced strict measures 4 weeks after the first COVID-19 death, which may have led to an initial surge in cases. However, mortality was decreased in the weeks subsequent to these strict measures, possibly leading to lower overall excess mortality. In Denmark, despite the lack of enforcement of measures with an SI ≥75%, the Danish government was among the first countries in Europe to act firmly against the virus by declaring a national lockdown and closing its borders, which, along with other social factors, was sufficient to prevent excess mortality.^39^

On the contrary, the lack of or only modest excess mortality in countries such as Austria, Estonia, Israel, Norway, Cyprus, Georgia, Slovenia and Ukraine can be partly attributed to the implementation of measures of stringency of ≥75% within 2 weeks of the first COVID-19 death in these countries. Previous studies gave evidence that a strict lockdown is associated with a rapid and large decrease in transmission as measured by the effective reproduction number.^40^^,^^41^ Our study adds to this evidence by suggesting that the introduction of strict lockdown measures in the early pandemic phase may also be associated with lower mortality.

Furthermore, the decreased mortality seen in some countries located in the tropical region or in the southern hemisphere, such as Colombia, Cape Verde and Australia, are most likely attributed to the different timing of the COVID-19 pandemic in these countries. The different seasonality patterns and different meteorological factors, coupled with strict control measures informed from lessons learned based on countries affected earlier on, led to a mild impact of the pandemic in these countries before July, thereby explaining their lack of excess mortality within the date window of this study. More specifically, Colombia experienced a steady increase in cases since March but had its peak of the COVID-19 pandemic in July and August 2020; Australia had a minor peak in coronavirus cases in March, but the number of cases substantially escalated in July 2020; and Cape Verde started seeing a surge in cases over the summer, but the peak of the pandemic was experienced in September–October 2020.^30^ Moreover, Australia was entering the influenza season as the restrictions were introduced and the government brought forward and expanded the flu-vaccination campaign. As a consequence, flu deaths were delayed or avoided, contributing to reduced all-cause mortality.

### Sex differences

In this study, some countries that did not display changes in mortality for the total population demonstrated sex-specific increases or decreases. Males in Israel and Ukraine and females in Ireland demonstrated increases in mortality. This highlights the importance of examining sex-specific differences in all-cause mortality, as there is an evident sex difference in excess mortality.^2^

### Challenges in COVID-19 and all-cause excess-mortality investigations

One challenge in excess-mortality investigations, which complicates between-country statistical comparisons, is the issue of delays in death registrations within and between countries, which is complicated even further by whether the country reports deaths by the date of death or by the date of registration. Delays in death reporting can range from a few days to a few weeks.^6^^,^^21^^,^^22^ We attempted to account for such delays by allowing a minimum of 4 weeks between the end of the observation period and data acquisition, and by carrying out a sensitivity analysis, which demonstrated that our results were not affected by reporting delays. However, death counts from each country may be differentially affected by delays in registrations, making comparisons up to one time point inaccurate.

Another challenge that contributes to the variability of results between countries is differential practices in death reporting. Many studies have previously established significant inaccuracies in the COD as reported on death certificates and the WHO estimates that only 13% of the world’s population resides in countries with ideal death-registration systems (reviewed in ^42^). These inaccuracies may have been exacerbated during the COVID-19 pandemic. More specifically, a report on the reporting of COVID-19 deaths in five European countries has identified regulatory and legislative differences in the procedures followed regarding the completion of death certificates.^43^ Differential practices between countries include, among others, the need for an external examiner, who is authorized to complete the death certificate, and the legislative requirement to notify authorities of suspected COVID-19 deaths. Additionally, regarding the COD, the report highlighted differences between countries in how this is documented on the death certificate since, in most countries, the medical practitioner completing the form can exert considerable discretion on what is listed on the certificate. Information provided on the causes of death and reported on death certificates was found to vary considerably, based on different conventions and/or rules regarding whether COVID-19 was included as a direct or indirect COD.

Furthermore, the different COVID-19-death definitions adopted by each country, but also the testing practices followed during the pandemic by different countries (testing only hospitalized patients, not testing care-home residents, etc.), adds uncertainty to investigations of COVID-19 and all-cause mortality.^44^^,^^45^

Also, depending on their geographical location, different countries experienced the first wave of the pandemic and its peak during different weeks or months of the year. Early in the pandemic, northern-hemisphere countries with cold climates appeared to be the most vulnerable to COVID-19 transmission, whereas southern-hemisphere countries and tropical regions seemed to be the least affected.^46^ As previously mentioned, these differences may be due to seasonality patterns and more specifically the role of meteorological factors affecting host susceptibility to infection and modes of transmission.^46^^,^^47^ The countries participating in this study have very diverse geographic locations, spanning both hemispheres. Even though we attempted to include in this publication data up until the end of August 2020, we were not able to fully capture the first wave of the pandemic; countries such as Cape Verde never experienced a peak in COVID-19 infections during the observation period of this study. Case numbers provide evidence that the COVID-19 pandemic only started to noticeably influence Cape Verde after June 2020.^48^ Therefore, the timing of such investigations needs to be considered when interpreting each country’s results.

Excess all-cause mortality investigations are made even more challenging by the evidenced differences in baseline mortality in different countries.^49^ In this study, the crude YTD all-cause mortality rate for countries providing data until August ranged from 353.1 deaths per 100 000 population in Israel to 874.7 deaths per 100 000 population in Georgia. Differences in baseline mortality between countries can be attributed to, among other things, the age distribution of populations, the different burdens of disease and differential access to healthcare.^49^ An appropriate measure to facilitate between-country comparisons is the P-Score, which takes the absolute difference in mortality as a percentage of the baseline mortality. However, even if excess mortality is presented with reference to baseline mortality, it is likely that baseline mortality and the reasons that contribute to a country’s baseline mortality can exaggerate or inflate excess mortality during the pandemic.

Finally, the strictness and timeliness of government measures, and geographical location as discussed in this study, are not the only potential contributors to excess mortality due to COVID-19. Baseline healthcare availability, access and quality in each country, and particularly a country's critical-care capacity and its ability to surge this capacity quickly under rising cases, were likely other contributors to excess mortality.^50^ On the other hand, country prosperity was shown to be associated with a higher COVID-19 spread and mortality for several reasons outlined elsewhere.^51^ Moreover, population density is associated with both COVID-19 infection and mortality.^52–54^ Therefore, variation in population densities between countries may also contribute to a variation in COVID-19 mortality and excess mortality. Also, the demographic and immunological profiles of populations, including their age structure, may also be impacting on their mortality experience during the COVID-19 pandemic.^55^ Undeniably, the first wave of the pandemic unfolded diverse and complex responses of citizens, governments and businesses. Civic behaviour with respect to social distancing and the use of masks, adequate contact-tracing services coupled with sufficient testing capacity and the smart use of mobile technologies, as well as community support and solidarity, along with good governance and tempered and evidenced-based government communication, are good examples of the complexity of effective response and resiliency to COVID-19.^56^ All the aforementioned complex factors probably synergistically or additively moderated mortality and contributed to the variability in mortality changes between countries. Albeit challenging and beyond the scope of the present study, a comprehensive analysis of the relative impact of these factors on COVID-19 and all-cause excess mortality is warranted.

### Strengths and limitations

Our study has some important strengths compared with other comparative mortality studies. First, it is one of the largest and most geographically diverse studies that relied on data from national and primary sources, rather than on publicly available data. Moreover, 19 out of the 22 countries (86.4%) included in the analysis were evaluated as having very high- or high-quality civil-registration and vital-statistics systems (only two and one countries were evaluated as of low and medium quality, respectively), reinforcing the validity of our results.^57^ In addition, it is one of the few studies investigating excess sex-specific mortality and also one of the few examining excess mortality in light of COVID-19 control measures. Lastly, our results were based on two independent methodologies, which demonstrated agreement in cases of increases or decreases in mortality. The use of two methodologies enabled us to investigate countries that lacked weekly mortality data and it also allowed us to validate Method 1: Observed 2020 versus 2015-2019 average mortality rate, which can be considered a simpler approach and feasible even for countries with mortality data of limited granularity, against Method 2: Observed versus expected 2020 deaths, which is based on a more accurate statistical methodology.

At the same time, our study also has limitations. First, for all the reasons outlined in the ‘Discussion’, our study did not attempt any between-country statistical comparisons or a pooled analysis, but focused instead on the mortality picture of each of the participating countries independently. Second, due to the lack of age-group-specific mortality data from many countries, the investigation of excess mortality by age group was not possible. Lastly, we cannot rule out that delays in death reporting may be affecting our results, even though we attempted to account for such delays by (i) allowing at least 4 weeks between the end of the observation period and data acquisition and (ii) performing a sensitivity analysis in which the observation period was truncated ≥3 months before data acquisition.

## Conclusion

In this excess-mortality investigation including 22 countries across the globe, it became evident that, up until the end of June or August 2020, several countries showed excess all-cause mortality compared with what was observed or expected based on the previous five years. Yet, other countries managed to avoid increases in all-cause mortality. The excess-mortality picture in the 22 participating countries was shown to be heavily influenced by the geographical location and seasonality of each country, as well as the promptness of governments to apply control measures of high stringency. As the pandemic continues and even worsens in many northern-hemisphere countries and as it now heavily affects countries of the southern hemisphere as well, the lessons learned from the first six or eight months of the pandemic can prove useful in order to minimize increases in all-cause mortality.

## Supplementary data

Supplementary data are available at *IJE* online.

## Supplementary Material

dyab123_Supplementary_Data
