# Supplementary material for: Excess all-cause mortality and COVID-19-related mortality: a temporal analysis in 22 countries, from January until August 2020
Source: Int J Epidemiol. 2021 Jul 20;51(1):35–53. doi: 10.1093/ije/dyab123 (PMC8344815; doi:10.1093/ije/dyab123)
Supplement: dyab123_Supplementary_Data [file dyab123_supplementary_data.zip › dyab123-suppl_data/ije-2021-02-0219-File018.docx]

**Supplementary Table S1 – Summary of national data sources, period of available mortality data, time unit, and COVID-19 deaths definition per country**

| **Country** | **Partners** | **All cause deaths (2015-2020)** | | | | | **COVID-19 deaths (2020)** | | | | | |  |  |
| --- | --- | --- | --- | --- | --- | --- | --- | --- | --- | --- | --- | --- | --- | --- |
|  |  | **Access date** | **Source** | **Public data (Y/N)** | **Link** | **Notes** | **Type of death** | **Access date** | **Source** | **Public data (Y/N)** | **Link** | **Notes** | **Time unit** | **Time frame (week/ month)** |
| **Australia** | Deakin University; Western Australia Department of Health; St John Ambulance Western Australia | 04/10/2020 | Australian Bureau of Statistics | Y | [Link](https://www.abs.gov.au/statistics/health/causes-death/provisional-mortality-statistics/latest-release) |  | Cause of death | 04/10/2020 | Australian Bureau of Statistics | Y | [Link](https://www.abs.gov.au/statistics/health/causes-death/provisional-mortality-statistics/latest-release) | Gender-specific COVID-19 deaths not available | National week counting | 26 |
| **Austria** | Medical University of Vienna | 05/10/2020 | Statistics Austria | Y | [Link](https://data.statistik.gv.at/web/meta.jsp?dataset=OGD_gest_kalwo_GEST_KALWOCHE_100) |  | Cause of death or contributing condition | 21/10/2020 | Austrian Agency for Health and Food Security | Y | [Link](https://covid19-dashboard.ages.at/dashboard_Tod.html?l=en) | Gender-specific COVID-19 deaths not available | ISO week | 35 |
| **Brazil** | Institute for Studies in Public Health, Federal University of Rio de Janeiro; Fluminense Federal University | 29/09/2020 | The Mortality Information System | Y | [Link](http://www2.datasus.gov.br/DATASUS/index.php?area=0901&item=1&acao=26&pad=31655) | 2015-2019 data | Cause of death or contributing condition | 29/09/2020 | The surveillance system of severe acute respiratory syndromes, Ministry of Health | Y | [Link](https://opendatasus.saude.gov.br/dataset/bd-srag-2020) |  | Epi week | 35 |
|  |  |  |  | Y | [Link](https://opendatasus.saude.gov.br/dataset/sistema-de-informacao-sobre-mortalidade) |  |  |  |  |  |  |  |  |  |
|  |  |  | Civil Registry | Y | [Link](https://github.com/capyvara/brazil-civil-registry-data) | 2020 data |  |  |  |  |  |  |  |  |
| **Cape Verde** | Jean Piaget University of Cape Verde | 01/09/2020 | Ministry of Health | Y | [Link](https://www.minsaude.gov.cv/index.php/documentosite/-1) |  | Cause of death or contributing condition | 01/09/2020 | Ministry of Health; Integrated surveillance system and emergency response; National Public Health Institute | N |  | Personal communication | Month | 6 |
| **Colombia** | Cooperative University of Colombia, campus Villavicencio | 08/2020 | Departamento Administrativo Nacional de estadistica | Y/N | [Link](http://www.dane.gov.co/) | Data only accessible within Colombia | Cause of death or contributing condition | 08/2020 | Instituto Nacional de Salud | Y/N | [Link](http://www.ins.gov.co/) | Data only accessible within Colombia | Trimester | 6 |
| **Cyprus** | University of Nicosia | 21/11/2020 | Eurostat | Y | [Link](https://ec.europa.eu/eurostat/databrowser/view/demo_r_mwk_ts/default/table?lang=en) |  | Cause of death | 21/11/2020 | Epidemiological Surveillance Unit, Ministry of Health | Y* | [Link](http://pio.gov.cy/coronavirus/categories/info%20&%20http:/pio.gov.cy/coronavirus/categories/press) | COVID-19 deaths announced on a daily basis | ISO week | 35 |
| **Denmark** | University of Copenhagen | 10/08/2020 | Statistics Denmark | Y | [Link](file:///C:\Users\jgabe\Documents\statbank.dk\dodc1) |  | Cause of death or contributing condition | 10/08/2020 | Statens Serum Institut | Y | [Link](https://www.ssi.dk/sygdomme-beredskab-og-forskning/sygdomsovervaagning/c/covid19-overvaagning) | Gender-specific COVID-19 deaths not available | Epi week | 35 |
| **England and Wales** | St George's, University of London; Imperial College London; Cardiff University | 24/09/2020 | Office for National Statistics | Y | [Link](https://www.ons.gov.uk/peoplepopulationandcommunity/birthsdeathsandmarriages/deaths/datasets/weeklyprovisionalfiguresondeathsregisteredinenglandandwales) | Datasets: "publishedweek2015"; "publishedweek2016" "publishedweek2017"; "publishedweek2018"; "publishedweek2019"; "publishedweek372020" | Cause of death | 24/09/2020 | Office for National Statistics | Y | Link | "publishedweek372020" | National week counting | 35 |
| **Estonia** | National Institute for Health Development | 31/07/2020 | National Institute for Health Development | Y/N | [Link](https://statistika.tai.ee/pxweb/en/Andmebaas/Andmebaas__01Rahvastik__04Surmad/?tablelist=true) | Weekly data are not published routinely, data for longer periods (month, year) are publicly available on website | Cause of death | 31/07/2020 | National Institute for Health Development | Y/N | [Link](https://statistika.tai.ee/pxweb/en/Andmebaas/Andmebaas__01Rahvastik__04Surmad/?tablelist=true) | Weekly data are not published routinely, data for longer periods (month, year) are publicly available on website | ISO week | 26 |
| **France** | French School of Public Health | 27/10/2020 | Public Health France | Y | [Link](https://www.data.gouv.fr/en/datasets/fichier-des-personnes-decedees/) |  | Cause of death | 27/10/2020 | National Institute of Demographic Studies/Public Health France | Y | [Link](https://dc-covid.site.ined.fr/fr/donnees/france/) | Only hospital and nursing homes COVID-19 deaths | ISO week | 35 |
| **Georgia** | Tbilisi State Unversity | 15/09/2020 | Vital Registration System of the National Center for Disease Control and Public Health | Y | [Link](https://www.geostat.ge/en) |  | Cause of death | 15/09/2020 | Vital Registration System of the National Center for Disease Control and Public Health National Statistics Office of Georgia | Y | [Link](https://www.geostat.ge/en) |  | ISO week | 35 |
|  |  |  | National Statistics Office of Georgia | Y | [Link](https://www.geostat.ge/en) |  |  |  |  |  |  |  |  |  |
| **Ireland** | Health Protection Surveillance Centre | 29/11/2020 | General Registers Office | N |  | Personal communication | Cause of death | 29/11/2020 | Health Protection Surveillance Centre | N |  | Personal communication | ISO week | 35 |
| **Israel** | The Israel Center for Disease Control Ministry of Health |  | Ministry of Health | N |  | Ministry of Health Personnel | Cause of death or contributing condition |  | Ministry of Health | N |  | Ministry of Health Personnel | Epi week | 35 |
| **Italy** | University of Perugia | 30/06/2020 | Italian National Institute of Statistics | Y | [Link](https://www.istat.it/it/archivio/240401) |  | Cause of death or contributing condition | 30/06/2020 | Civil Protection, Ministry of Health | Y | [Link](https://gisanddata.maps.arcgis.com/apps/opsdashboard/index.html#/b0c68bce2cce478eaac82fe38d4138b1) | Gender-specific COVID-19 deaths not available | Epi week | 26 |
| **Northern Ireland** | St George's, University of London Imperial College London | 22/09/2020 | Northern Ireland Statistics and Research Agency | Y | [Link](https://www.nisra.gov.uk/publications/weekly-deaths) | "Historical Weekly Deaths, 2011-2019" | Cause of death | 22/09/2020 | Northern Ireland Statistics and Research Agency | Y | [Link](https://www.nisra.gov.uk/publications/weekly-deaths) | Dataset "Weekly deaths - ending 11 September 2020"; | National week counting | 35 |
|  |  | 23/10/2020 |  |  | [Link](https://www.nisra.gov.uk/sites/nisra.gov.uk/files/publications/Weekly%20Deaths%20by%20Age%20and%20Respiratory%20Deaths%2C%202011-2019.xls) | Supplementary historical data |  |  |  |  |  |  |  |  |
| **Norway** | University of Oslo | 10/07/2020 & 23/09/2020 | Statistics Norway | Y | [Link](https://www.ssb.no/statbank/table/07995/) |  | Cause of death or contributing condition | 05/10/2020 | Norwegian Surveillance System for Communicable Diseases | N | [Link](https://www.fhi.no/en/hn/health-registries/msis/) | Data available for certified research institutions upon official data request; Gender-specific COVID-19 deaths not available | ISO week | 35 |
| **Scotland** | St George's, University of London Imperial College London | 10/05/2020 | National Records of Scotland | Y/N | [Link](https://www.nrscotland.gov.uk/statistics-and-data/statistics/statistics-by-theme/vital-events/general-publications/weekly-and-monthly-data-on-births-and-deaths/weekly-data-on-births-and-deaths) | Data entitled 'deaths-time-series-19-dt.4 by sex and age yearly data'. | Cause of death | 24/09/2020 | National Records of Scotland | Y | [Link](https://www.nrscotland.gov.uk/statistics-and-data/statistics/statistics-by-theme/vital-events/general-publications/weekly-and-monthly-data-on-births-and-deaths/deaths-involving-coronavirus-covid-19-in-scotland) |  | ISO week | 35 |
|  |  | 19/10/2020 |  |  |  | 2019 data for respiratory deaths not available; supplementary data detailing respiratory deaths 2015-2018 provided by email. This data will be publicly available when it includes the 2019 data. |  |  |  |  |  |  |  |  |
| **Slovenia** | University of Ljubljana | 06/10/2020 | National Institute of Public Health | N | [Link](https://www.euromomo.eu/graphs-and-maps) | Personal communication | Cause of death or contributing condition | 23/11/2020 | National Institute of Public Health | N |  | Personal communication | ISO week | 35 |
| **Spain** | University of Oviedo | 08/12/2020 | Instituto de Salud Carlos III (MoMo System) | Y | [Link](https://momo.isciii.es/public/momo/dashboard/momo_dashboard.html) | Gender-specific all-cause mortality not available | Cause of death or contributing condition | 08/12/2020 | Ministerio de Sanidad | Y | [Link](https://www.mscbs.gob.es/profesionales/saludPublica/ccayes/alertasActual/nCov/situacionActual.htm) | Gender-specific COVID-19 deaths not available | Epi week | 35 |
|  |  |  |  |  |  |  |  |  | Instituto Nacional de Estadística | Y | [Link](https://www.ine.es/covid/covid_salud.htm) |  |  |  |
| **Sweden** | Karolinska Institute | 18/09/2020 | Statistics Sweden | Y | [Link](https://www.scb.se/om-scb/nyheter-och-pressmeddelanden/folj-preliminar-statistik-om-dodsfall/) | Website for overall number of deaths and sex-specific data between 2015-2020 | Cause of death | 16/10/2020 | National Board of Health and Welfare | Y/N | [Link](https://www.socialstyrelsen.se/statistik-och-data/statistik/statistik-om-covid-19/statistik-over-antal-avlidna-i-covid-19/) | Website for overall number of deaths; Personal communication for sex-specific data | Month | 8 |
| **Ukraine** | Bogomolets National Medical University | 11/11/2020 | State Statistics Service of Ukraine | Y | [Link](http://database.ukrcensus.gov.ua/MULT/Dialog/statfile_c_files/az.html) |  | Cause of death | 11/11/2020 | Cabinet of Ministers of Ukraine | Y/N | [Link](https://covid19.gov.ua/analitichni-paneli-dashbordy) | Personal communication for data validation | ISO week | 35 |
| **USA** | University of Texas Medical Branch; University of South Carolina | 14/10/2020 | Human Mortality Database. University of California, Berkeley (USA), and Max Planck Institute for Demographic Research (Germany) | Y | [Link](https://www.mortality.org/) | Data available upon request access to the database. Data available until week 5 2020 | Cause of death or contributing condition | 14/10/2020 | National Center for Health Statistics | Y | [Link](https://data.cdc.gov/NCHS/Provisional-COVID-19-Death-Counts-by-Sex-Age-and-W/vsak-wrfu) | Data available since epi week 5 in 2020 | Epi week | 35 |
|  |  |  | National Center for Health Statistics | Y | [Link](https://data.cdc.gov/NCHS/Provisional-COVID-19-Death-Counts-by-Sex-Age-and-W/vsak-wrfu) | Data available since Epi week 5 2020 |  |  |  |  |  |  |  |  |

Abbreviations: ISO: International Organization for Standardization; Epi: epidemiological
