# Supplementary figures and images for: Excess all-cause mortality and COVID-19-related mortality: a temporal analysis in 22 countries, from January until August 2020
Source: Int J Epidemiol. 2021 Jul 20;51(1):35–53. doi: 10.1093/ije/dyab123 (PMC8344815; doi:10.1093/ije/dyab123)

**Supplementary Figure S1 – Map highlighting the 22 countries contributing data to this study**


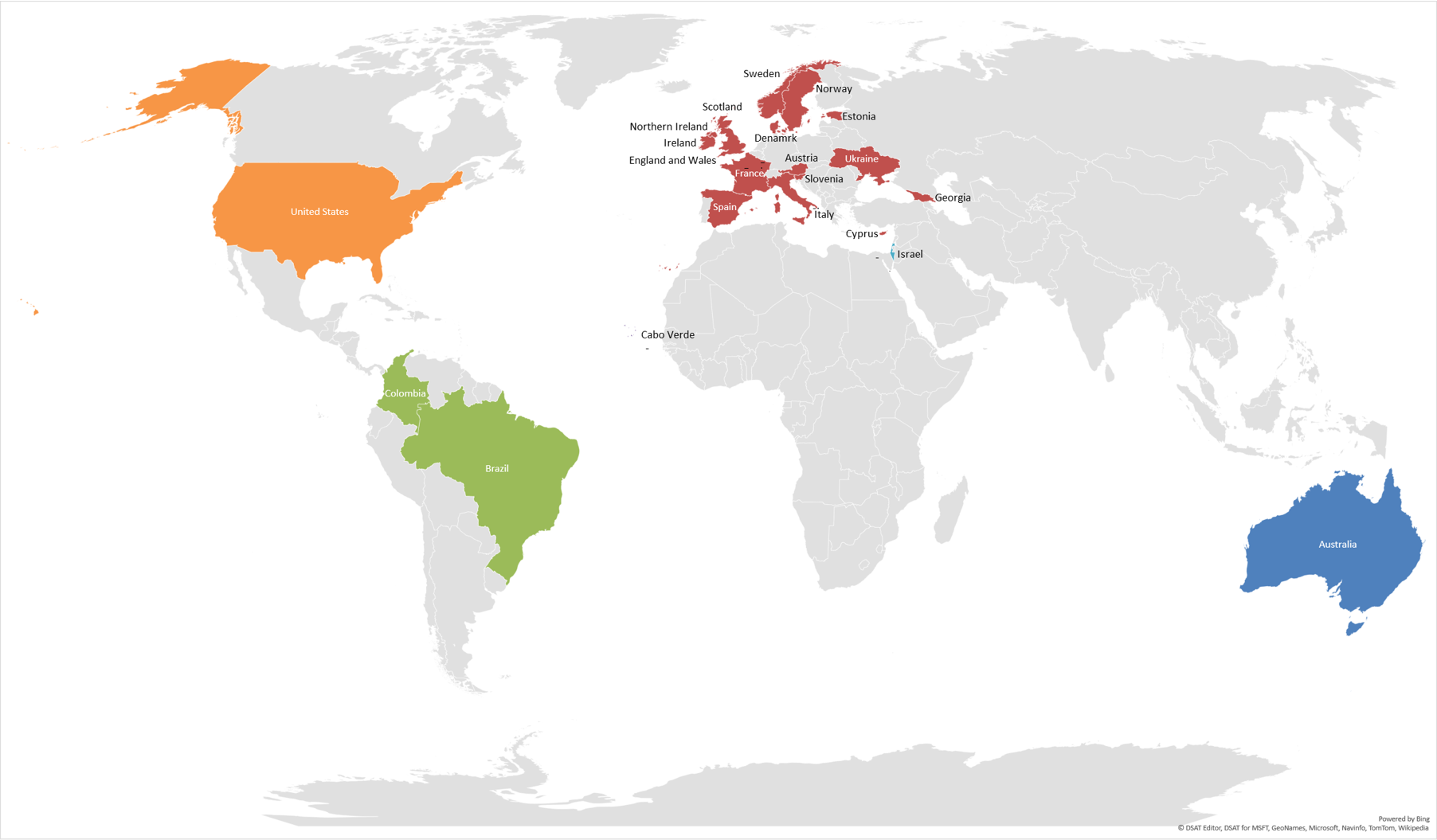

Supplement: dyab123_Supplementary_Data [file dyab123_supplementary_data.zip › dyab123-suppl_data/ije-2021-02-0219-File016.docx]

**Supplementary Figure S2 – Graphs of excess deaths and COVID-19 attributed deaths**


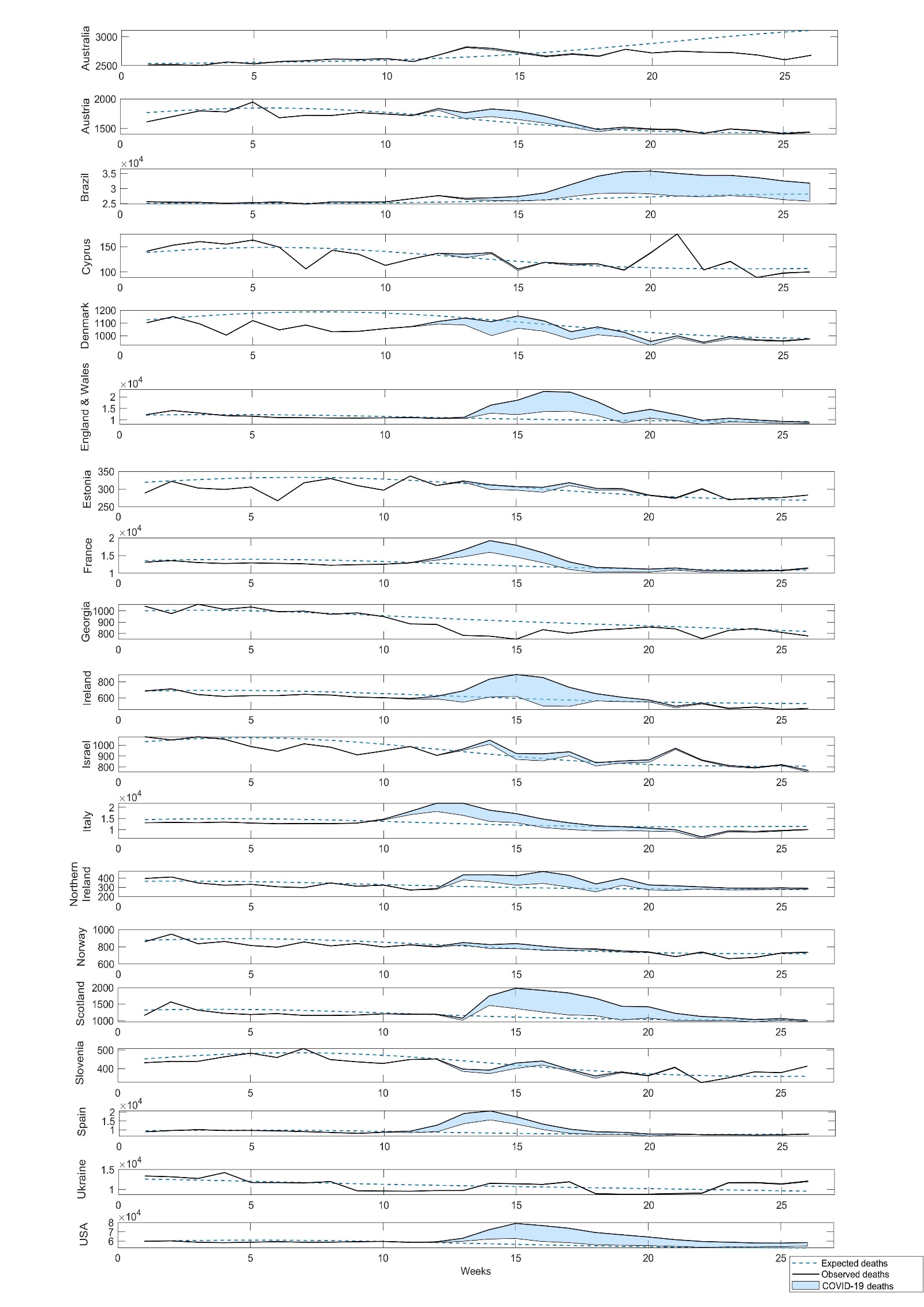

Supplement: dyab123_Supplementary_Data [file dyab123_supplementary_data.zip › dyab123-suppl_data/ije-2021-02-0219-File017.docx]
